# Posture management for neck pain: identification and quantification of associated factors based on a multicentre cross-sectional study in China

**DOI:** 10.7189/jogh.16.04240

**Published:** 2026-08-21

**Authors:** Guangqi Lu, Hanze Mao, Xinran Chen, Xinyue Sun, Yakun Liu, Mingming Ma, Jing Li, Shuaiqi Zhou, Jiaming Hu, Guoliang Ma, Zhizhuang Wang, Xin Xiu, Minghui Zhuang, Bin Tang, Liguo Zhu, Jie Yu

**Affiliations:** 1 Wangjing Hospital, China Academy of Chinese Medical Sciences, Beijing, China; 2 Center for Applied Statistics, School of Statistics, Renmin University of China, Beijing, China

**Keywords:** neck pain, associated factors, posture, electronic devices, cross-sectional study

## Abstract

**Background:**

Neck pain (NP) is a leading musculoskeletal disorder globally. Posture management is crucial for its prevention; however, population-level evidence quantifying the associated factors in real-world settings remains limited. We aimed to identify and quantify threshold effects of key factors related to posture management associated with NP among Chinese adults.

**Methods:**

We conducted a multicentre, cross-sectional study across six Chinese cities. We collected data *via* face-to-face interviews using a questionnaire covering demographics, occupational factors, electronic device use, sleep, transportation, and exercise. Multivariable logistic regression identified factors associated with NP. We used restricted cubic splines to model nonlinear relationships, and applied the Akaike information criterion to determine dichotomisation cutoffs for continuous variables. Propensity score matching assessed the robustness of these associations, and subgroup analyses by sex and age assessed heterogeneity.

**Results:**

We included a total of 6,969 adults (≥18 years), comprising 2,662 (38.2%) with NP and 4,307 (61.8%) without. Identified associated factors included: computer use time for work ≥2 hours/day (odds ratio (OR) = 1.39; 95% confidence interval (CI) = 1.16–1.67, *P* < 0.001), smartphone use time for work ≥3 hours/day (OR = 1.21; 95% CI = 1.03–1.42, *P* = 0.022), smartphone screen height below horizontal eye level (OR = 1.16; 95% CI = 1.00–1.34, *P* = 0.043), and firm mattress (OR = 1.19; 95% CI = 1.04–1.37, *P* = 0.012) were associated with increased odds of NP. Sleeping time ≥7 hours/day (OR = 0.79; 95% CI = 0.69–0.89, *P* < 0.001), supine sleeping (OR = 0.87; 95% CI = 0.76–0.99, *P* = 0.039) and walking as the primary mode of transportation (OR = 0.80, 95% CI = 0.70–0.91, *P* < 0.001) were associated with decreased odds of NP.

**Conclusions:**

This study identified and quantified threshold effects of key factors associated with NP among Chinese adults, including electronic device use, sleep habits, and commuting patterns. These quantified thresholds offer actionable benchmarks for posture management of NP in clinical practice and public health interventions.

**Registration:**

Chinese Clinical Trial Registry: ChiCTR2400093567

Neck pain (NP) is a leading musculoskeletal disorder in adults worldwide [1]. According to the Global Burden of Disease Study 2021 [2], approximately 203 million people suffered from NP in 2020, a number projected to rise to 269 million by 2050 due to increasing electronic device use, changing lifestyles, and population ageing. China bears one of the highest burdens worldwide, with an estimated 49.9 million cases in 2020, an increase of over 80% in the past three decades. Beyond its high prevalence, NP imposes substantial personal, economic, and societal burdens: it impairs daily functioning, limits physical activity, reduces work productivity, and contributes significantly to healthcare expenditures. These consequences underscore the urgent need for effective prevention and management strategies.

Posture management in daily life and work is crucial for preventing NP in the digital era, with factors such as smartphone use [3,4], sleep posture [5], and commuting habits [6] having been shown to contribute to cervical load. However, most existing evidence comes from single-centre studies with small sample sizes and merely identifies associations without quantifying clinically actionable thresholds. Moreover, population-level evidence quantifying these associated factors in real-world, multicentre settings remains limited.

Our primary aim was to identify and quantify threshold effects of key factors associated with NP among Chinese adults, with particular focus on electronic device use, occupational postures, and daily lifestyle habits, to inform posture management strategies for NP.

## METHODS

We employed restricted cubic splines (RCS), Akaike information criterion (AIC), propensity score matching (PSM), and subgroup analyses by sex and age to address these gaps. We conducted this cross-sectional study between March and November 2025.

### Participants

We included adults aged 18 years or older who had been living for at least six months in six major cities in China: Beijing, Shenzhen, Changchun, Xi’an, Changsha, and Suzhou. We excluded individuals with a history of cervical trauma, fracture, or dislocation, severe cervical deformity, tumour, tuberculosis, or ankylosing spondylitis, bedridden status with inability to perform daily living activities, and those with diagnosed psychiatric or cognitive disorders that could impede comprehension or reliable response.

We estimated the sample size using the standard formula for cross-sectional studies: N = deff × [Z^2^α/2 × π(1-π)]/δ^2^, where deff (design effect) was set at 1.5 to account for the multi-stage sampling design, α at 0.05 (two-sided), Zα/2 at 1.96, π (expected prevalence) at 13.76% based on a prior study about cervical spondylosis in Chinese adults [7], and δ (margin of error) at 1%. The calculated minimum sample size was 6,838.

We implemented a three-stage stratified random sampling design. At each of the first two stages, sampling units were selected using a computer-generated random number algorithm applied to complete sampling frames. In the first stage, we selected districts based on city population size; four urban districts were randomly selected from Beijing, while two urban districts were randomly selected from each of the other five cities. In the second stage, we randomly selected five residential communities from each of the 14 districts identified in the first stage. In the final stage, we targeted 100 participants for recruitment from each selected community. We defined NP as self-reported pain lasting at least 24 hours in the cervical region (C1–C7) within the preceding three months.

### Data collection

We collected data through face-to-face interviews using a standardised electronic questionnaire, designed to capture information on posture-related behaviours associated with NP. The questionnaire covered demographics: age, sex, and educational attainment; occupational factors: daily working hours, head-down time (total daily duration and duration per session), mental and physical workload intensity, and duration of maintaining the same posture per session; electronic device use: daily duration of computer and smartphone use (separately for work and entertainment), and screen height relative to eye level; transportation: primary mode of transportation, daily travel time, bag type and weight; sleep: daily sleep duration, sleeping positions, pillow height, and mattress firmness; and exercise: exercise frequency and duration per session (**Table 1**; **Online Supplementary Document**).

**Table 1:** Comparison of characteristics between participants with and without neck pain*

	Neck pain (n = 2.662)	No neck pain (n = 4.307)	*P*-value
**Sex**			<0.001
Male	935 (35.1)	2,026 (47.0)	
Female	1,727 (64.9)	2,281 (53.0)	
**Age in years, Mdn (IQR; range)**	41.0 (27.0–54.0; 18.0–95.0)	40.0 (25.0–55.0; 18.0–95.0)	0.009
**BMI, kg/m^2^, Mdn (IQR; range)†**	23.4 (20.9–26.0; 14.0–49.1)	23.4 (21.0–25.9; 13.6–53.3)	0.752
**Educational attainment**			<0.001
High school or below	857 (32.2)	1,540 (35.8)	
Bachelor's degree or junior college	1,219 (45.8)	2,180 (50.6)	
Master's degree or above	586 (22.0)	587 (13.6)	
**Keeping head down time per day in hours, Mdn (IQR; range)‡**	5.0 (3.0–7.0; 0–15.0)	4.0 (2.0–6.0; 0–15.0)	<0.001
**Keeping head down time once in hours, Mdn (IQR; range)§**	0.5 (0.3–1.0; 0.1–8.0)	0.5 (0.3–0.8; 0.1–8.0)	<0.001
**Working time per day in hours, Mdn (IQR; range)**	8.0 (6.0–9.0; 0–15.0)	8.0 (6.0–8.0; 0–16.0)	<0.001
**Maintaining the same working posture time once in hours, Mdn (IQR; range)**	1.0 (0.5–2.0; 0–10.0)	1.0 (0.5–2.0; 0–10.0)	<0.001
**Mental workload intensity‖**			<0.001
Light	752 (28.3)	1,536 (35.7)	
Moderate	1,331 (50.0)	2,133 (49.5)	
High	579 (21.7)	638 (14.8)	
**Physical work intensity‖**			0.512
Light	1,454 (54.6)	2,312 (53.7)	
Moderate	1,092 (41.0)	1,815 (42.1)	
High	116 (4.4)	180 (4.2)	
**Use of computer**			<0.001
Yes	1,526 (57.3)	1,778 (41.3)	
No	1,136 (42.7)	2,529 (58.7)	
**Computer use time for work per day in hours, Mdn (IQR; range)**	2.0 (0–6.0; 0–14.0)	0 (0–3.0; 0–15.0)	<0.001
Computer use time for entertainment per day in hours, Mdn (IQR; range)*	0 (0–1.0; 0–12.0)	0 (0–1.0; 0–13.0)	<0.001
**Computer screen height**			0.757
Above horizontal eye level	427 (28.0)	483 (27.2)	
At horizontal eye level	675 (44.2)	805 (45.3)	
Below horizontal eye level, within 45°	398 (26.1)	450 (25.3)	
Below horizontal eye level, exceeding 45°	26 (1.7)	40 (2.2)	
**Use of smartphone**			<0.001
Yes	2,124 (79.8)	2,668 (62.0)	
No	538 (20.2)	1,639 (38.0)	
**Smartphone use time for work per day in hours, Mdn (IQR; range)**	2.0 (0–5.0; 0–13.0)	1.0 (0–4.0; 0–15.0)	<0.001
**Smartphone use time for entertainment per day in hours, Mdn (IQR; range)**	2.0 (1.0–4.0; 0–15.0)	1.0 (0–3.0; 0–16.0)	<0.001
**Smartphone screen height**			<0.001
Above horizontal eye level	245 (11.5)	382 (14.3)	
At horizontal eye level	472 (22.2)	614 (23.0)	
Below horizontal eye level, within 45°	1,136 (53.5)	1,414 (53.0)	
Below horizontal eye level, exceeding 45°	271 (12.8)	258 (9.7)	
**Sleeping time per day in hours, Mdn (IQR; range)**	7.0 (6.0–8.0; 5.0–13.0)	7.0 (6.0–8.0; 5.0–12.0)	<0.001
**Sleeping position**			
Supine	1,471 (55.3)	2,746 (63.8)	<0.001
Prone	225 (8.5)	384 (8.9)	0.506
Left lateral decubitus	1,022 (38.4)	1,404 (32.6)	<0.001
Right lateral decubitus	1,356 (50.9)	1,836 (42.6)	<0.001
**Pillow height, cm**			<0.001
No pillow	83 (3.1)	280 (6.5)	
5–10	1,808 (67.9)	2,885 (67.0)	
10–20	691 (26.0)	1,024 (23.8)	
≥20	80 (3.0)	118 (2.7)	
**Mattress firmness¶**			<0.001
Soft	185 (6.9)	428 (9.9)	
Medium	1,653 (62.1)	2,672 (62.0)	
Firm	824 (31.0)	1,207 (28.0)	
**Primary mode of transportation**			<0.001
Walking	881 (33.1)	1,812 (42.1)	
Cycling	498 (18.7)	753 (17.5)	
Driving	580 (21.8)	815 (18.9)	
Passenger	703 (26.4)	927 (21.5)	
**Travel time per day in hours, Mdn (IQR; range)**	0.5 (0.3–1.0; 0.1–3.0)	0.5 (0.3–1.0; 0.1–3.0)	0.438
**Carry a bag**			<0.001
Yes	1,550 (58.2)	1,936 (45.0)	
No	1,112 (41.8)	2,371 (55.0)	
**Type of bag**			
Handbag	162 (10.5)	173 (8.9)	<0.001
Single-strap shoulder bag	555 (35.8)	588 (30.4)	<0.001
Crossbody bag	436 (28.1)	467 (24.1)	<0.001
Backpack	666 (43.0)	949 (49.0)	0.004
**Bag weight, kg**			<0.001
No bag	1,112 (41.8)	2,371 (55.0)	
<1	679 (25.5)	905 (21.0)	
1–5	812 (30.5)	908 (21.1)	
≥5	59 (2.2)	123 (2.9)	
**Exercising frequency per week**			0.471
No exercise	651 (24.5)	1,128 (26.2)	
1–3	1,008 (37.9)	1,460 (33.9)	
3–5	530 (19.9)	865 (20.1)	
≥5	473 (17.8)	854 (19.8)	
**Exercising time once in hours, Mdn (IQR; range)**	0.5 (0.2–1.0; 0–10.0)	0.5 (0–1.0; 0–10.0)	0.001

BMI – body mass index, IQR – interquartile range, Mdn – median

*Presented as n (%) unless specified otherwise.

†Height and weight were measured using standardised instruments, and BMI was calculated as weight (kg) divided by height squared (m^2^).

‡Keeping head down time per day referred to the total cumulative duration across all sessions.

§Keeping head down time once referred to the duration of a single continuous session of maintaining a head-down posture before any break or posture change.

‖Mental and physical work intensity were each self-categorised by participants as ‘light, moderate, or high’ based on single-item global ratings of their main job.

¶Mattress firmness was assessed by a single self-report question, with response options anchored by behavioural descriptors: soft (body sinks considerably into the mattress), medium (moderately firm), or firm (body does not conform well to the mattress surface).

### Quality control

We developed the study questionnaire and protocols through multiple rounds of expert discussion and consensus to ensure scientific rigour and alignment with study objectives. We conducted a pilot test (n = 100) to evaluate the clarity and reliability of the questionnaire before the main survey. We revised items based on feedback to minimise subjectivity and inconsistency in self-reported data. Fieldwork was coordinated by a central lead centre, Wangjing Hospital, China Academy of Chinese Medical Sciences, Beijing, in collaboration with five participating clinical centres located in the other study cities. Each site appointed a site principal investigator, a team leader, and a dedicated quality control officer. All field staff underwent standardised training before the study. Competency was assessed through face-to-face evaluations conducted by the study leaders; only staff who passed these assessments were permitted to collect data. Before data collection, participants received a standardised explanation of the study objectives, procedures, and relevant information to ensure informed consent and build rapport. Clear instructions were provided and recall periods were kept short to reduce memory-related bias.

### Statistical analysis

We assessed continuous variables for normality using the Shapiro–Wilk test, and presented non-normally distributed data as medians (interquartile ranges) and compared using the Mann-Whitney U test. We presented categorical data as frequencies and percentages and compared them using the χ^2^ test or Fisher exact test. We presented ordinal data as frequencies and percentages and compared them using the Wilcoxon rank-sum test. Univariable analysis was performed for all candidate variables (**Table 1**). Variables with *P* < 0.05 in the univariable analysis were then entered into a multivariable logistic regression model. We presented results as adjusted odds ratios (ORs) with 95% confidence intervals (CIs). RCS with four knots (5th, 35th, 65th, and 95th percentiles) were used to model nonlinear dose-response relationships for behavioural continuous variables that were significantly associated with NP in the multivariable logistic regression, as associations between such behaviours and NP may not be linear. We determined dichotomisation cutoffs for continuous variables based on the AIC, which balances model fit against complexity and avoids arbitrary threshold selection. We performed PSM for each binary behavioural factor identified as significant in the multivariable logistic regression, using 1:1 nearest-neighbour matching with a calliper of 0.02. We assessed covariate balance using standardised mean differences (SMD), with SMD<0.10 indicating adequate balance. In the matched cohorts, we re-evaluated associations using multivariable logistic regression. We conducted subgroup analyses to explore heterogeneity across strata defined by sex and age. We included multiplicative interaction terms (risk factor × subgroup indicator) in the multivariable logistic regression models, and the statistical significance of each interaction term was assessed using the Wald test. We considered a two-sided *P*-value <0.05 statistically significant.

We performed the analysis from 2025 to 2026, and the Centre for Applied Statistics, School of Statistics, Renmin University of China performed all statistical analyses. We performed all analyses using *R*, version 4.4.0 (R Core Team, Vienna, Austria).

## RESULTS

We included a total of 6,969 participants, comprising 2,662 with NP (38.2%) and 4,307 without NP (61.8%) (**Table 1**).

### Multivariable logistic regression analysis

**Figure 1:**
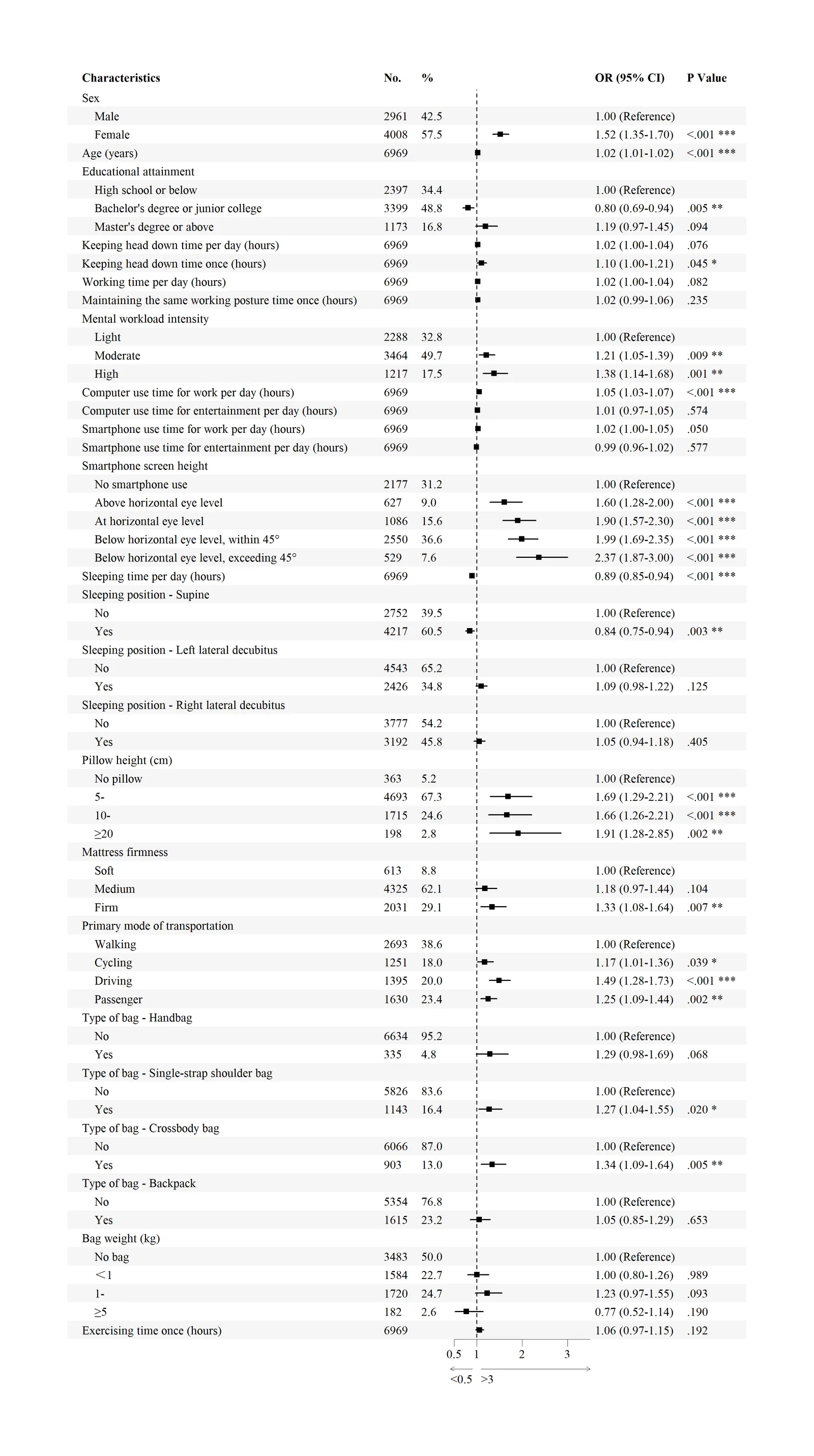
Multivariable logistic regression analysis of factors associated with neck pain. CI – confidence interval, OR – odds ratio.

We found that being female (*vs.* male), older age, longer keeping head down time once, higher mental workload intensity, longer computer use time for work per day, lower smartphone screen height, higher pillow height, firm mattress (*vs.* soft), non-walking transportation modes (*vs.* walking), and carrying a single-strap shoulder or crossbody bag were associated with increased odds of NP. Furthermore, we found that bachelor’s degree or junior college (*vs.* high school or below), supine sleeping position, and longer sleeping time per day were associated with decreased odds of NP (**Figure 1**).

### RCS analysis and AIC-based cutoffs

RCS analysis revealed nonlinear relationships for continuous variables. The RCS curves for each continuous variable are presented in Figures S1–4 in the **Online Supplementary Document**. Based on AIC, cutoffs for dichotomisation were determined as follows: 0.5-hour for keeping head down time once, 2 hours for computer use time for work per day, 3 hours for smartphone use time for work per day, and 7 hours for sleeping time per day.

### PSM analysis

PSM analysis confirmed that computer use time for work ≥2 hours/day (OR = 1.39; 95% CI = 1.16–1.67, *P* < 0.001), smartphone use time for work ≥3 hours/day (OR = 1.21; 95% CI = 1.03–1.42, *P* = 0.022), smartphone screen height below horizontal eye level (OR = 1.16; 95% CI = 1.00–1.34, *P* = 0.043), and firm mattress (OR = 1.19; 95% CI = 1.04–1.37, *P* = 0.012) were associated with increased odds of NP. Sleeping time ≥7 hours/day (OR = 0.79; 95% CI = 0.69–0.89, *P* < 0.001), supine sleeping (OR = 0.87; 95% CI = 0.76–0.99, *P* = 0.039) and walking as the primary mode of transportation (OR = 0.80; 95% CI = 0.70–0.91, *P* < 0.001) were associated with decreased odds of NP. Three factors that were significant in the multivariable logistic regression analysis – a keeping head down time once (≥0.5 hours), single-strap shoulder bag use, and crossbody bag use – did not remain significant after PSM, suggesting that these associations are less robust (**Table 2**).

**Table 2:** PSM analysis of factors associated with neck pain

Characteristics	Matched pairs, n	OR (95% CI)	*P*-value
Keeping head down time once in hours (≥0.5 *vs.* <0.5)	2,272	1.08 (0.95–1.22)	0.300
Computer use time for work per day in hours (≥2 *vs.* <2)	1,053	1.39 (1.16–1.67)	<0.001
Smartphone use time for work per day in hours (≥3 *vs.* <3)	1,342	1.21 (1.03–1.42)	0.022
Smartphone screen height (below horizontal eye level *vs.* at or above horizontal eye level)	1,613	1.16 (1.00–1.34)	0.043
Sleeping time per day in hours (≥7 *vs.* <7)	2,176	0.79 (0.69–0.89)	<0.001
Sleeping position – supine (yes *vs.* no)	1,917	0.87 (0.76–0.99)	0.039
Pillow height (no pillow *vs.* pillow)	321	0.74 (0.50–1.10)	0.130
Mattress firmness (firm *vs.* soft or medium firmness)	2,000	1.19 (1.04–1.37)	0.012
Primary mode of transportation (walking *vs.* other modes of transportation)	2,260	0.80 (0.70–0.91)	<0.001
Type of bag – single-strap shoulder bag (yes *vs.* no)	272	1.03 (0.70–1.51)	0.900
Type of bag – crossbody bag (yes *vs.* no)	265	1.31 (0.89–1.95)	0.200

CI – confidence interval, OR – odds ratio

### Subgroup analysis

For smartphone screen height below horizontal eye level, the association with NP strengthened with age (*P*-value for interaction = 0.005): OR = 1.28; 95% CI = 1.10–1.48 at 18–44 years; OR = 1.44; 96% CI = 1.17–1.78 at 45–64 years, and OR = 1.53; 95% CI = 1.06–2.22 at ≥65 years. Firm mattress use was associated with higher NP odds only in females (OR = 1.28; 95% CI = 1.11–1.49; *P*-value for interaction = 0.018), but not in males (OR = 1.02; 95% CI = 0.85–1.22). Walking as the primary mode of transportation was associated with lower NP odds only in males (OR = 0.61; 95% CI = 0.50–0.74; *P*-value for interaction = 0.002), but not in females (OR = 0.92; 95% CI = 0.80–1.06) (**Table 3**; Table S1 and Figures S5–11 in the **Online Supplementary Document**).

**Table 3:** Subgroup analysis of factors associated with neck pain*

	n	OR (95% CI)	*P*-value	*P*-value for interaction
**Smartphone screen height below horizontal eye level**				0.005
Age in years				
*18–45*	4,072	1.28 (1.10–1.48)	0.001	
*45–65*	2,105	1.44 (1.17–1.78)	<0.001	
*≥65*	792	1.53 (1.06–2.22)	0.023	
**Firm mattress**				0.018
Sex				
*Male*	2,961	1.02 (0.85–1.22)	0.849	
*Female*	4,008	1.28 (1.11–1.49)	<0.001	
**Walking as the primary mode of transportation**				0.002
Sex				
*Male*	2,961	0.61 (0.50–0.74)	<0.001	
*Female*	4,008	0.92 (0.80–1.06)	0.260	

CI – confidence interval, OR – odds ratio

*Only factors with significant interaction (*P*-value for interaction <0.05) are shown. Complete subgroup analysis results for all factors are presented in Table S1, Figures S5–11 in the **Online Supplementary Document**.

## DISCUSSION

**Figure 2:**
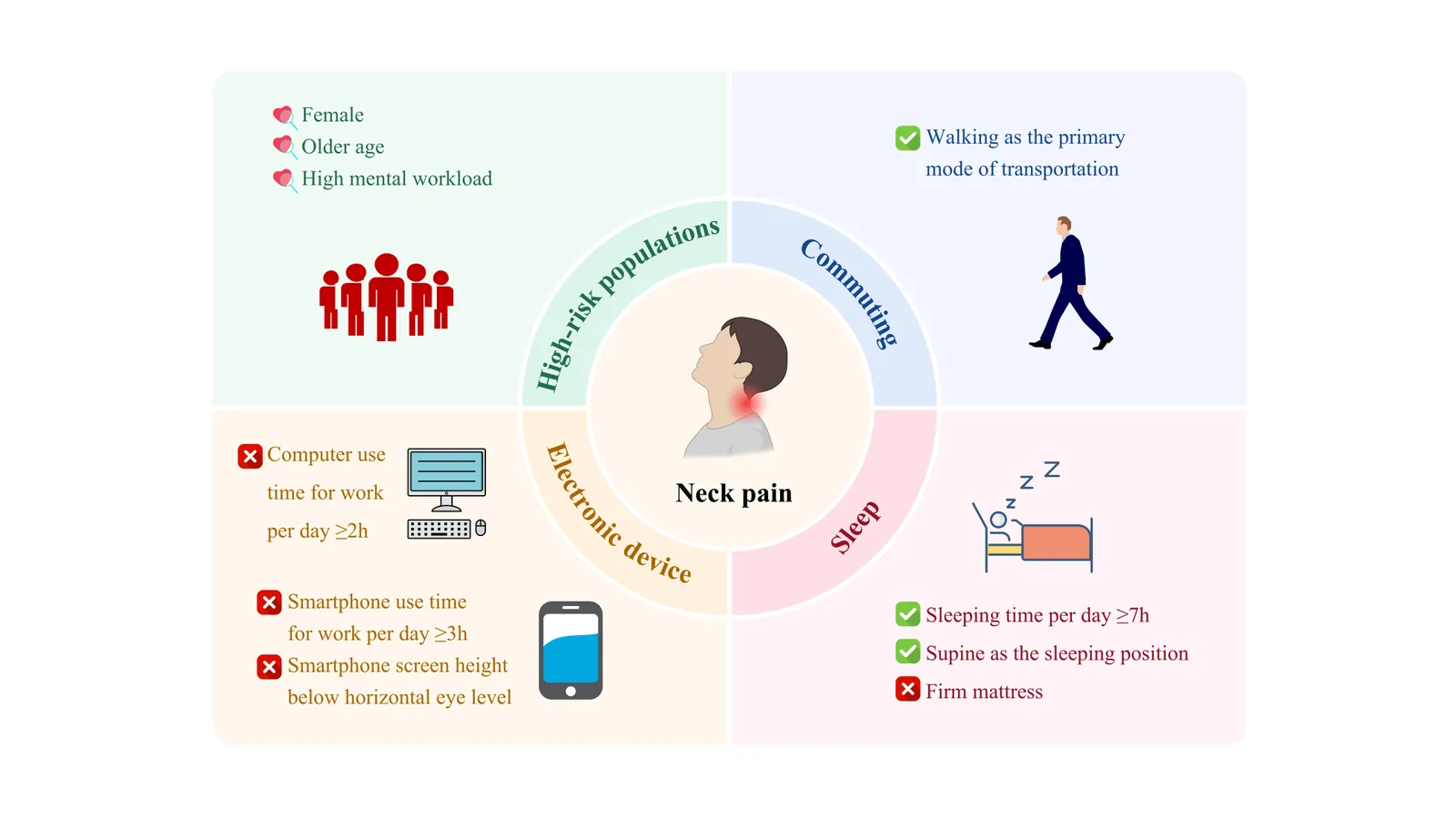
A summary of identified factors associated with neck pain.

Here, we provide population-based evidence on factors associated with NP among Chinese adults, with contributions: identifying key posture-related factors (electronic device use, sleep habits, and commuting patterns), quantifying threshold effects (computer use time for work ≥2 hours/day, smartphone use time for work ≥3 hours/day, sleep time ≥7 hours/day) (**Figure 2**).

Prolonged work-related computer and smartphone use, along with lower smartphone screen position, were positively associated with NP. These associations may be explained by forward head posture, which increases neck flexion angle and contributes to sustained static load on cervical muscles [8–10]. The mechanical load increases progressively with greater neck flexion angles [11,12]. Our analysis identified thresholds of 2 hours/day for work-related computer use and 3 hours/day for work-related smartphone use. The identified thresholds may correspond to the point at which sustained static muscle contraction begins to induce significant muscle fatigue and discomfort. The observed thresholds for screen time associated with NP vary across studies [13–16], likely due to differences in study populations, age groups, device types, and analytical methods. The association between smartphone screen height below horizontal eye level and NP strengthened with age. This may reflect age-related structural changes in the cervical spine, such as disc dehydration, osteophyte formation, and facet joint degeneration [17,18], which reduce load tolerance and may increase susceptibility to sustained postural load. Notably, work-related electronic device use was associated with NP, whereas entertainment use was not. One possible explanation is that cognitive demands in work settings may lead to more rigid, sustained postures [19], potentially increasing cumulative mechanical load [20]. In contrast, recreational use may involve more frequent posture shifts. However, this hypothesis requires testing in future studies with objective posture monitoring.

Longer sleep duration, particularly exceeding seven hours, was significantly associated with lower odds of NP, consistent with previous findings [21,22]. The underlying mechanisms likely involve the critical roles of sleep in tissue repair and pain modulation [23,24]. The cervical intervertebral disc exhibits a biomechanically mediated circadian rhythm. Insufficient sleep disrupts this circadian pattern, thereby accelerating disc degeneration [25–27]. While few population-based studies have examined the association between sleep posture and NP, our findings suggest that supine sleeping may be protective, possibly due to its facilitation of neutral cervical alignment. The association between firm mattress use and NP may be due to its inability to conform to the spine’s natural curvature. Particularly in lateral positions, this may lead to non-physiological spinal alignment, which can transmit compensatory stresses to the cervical region [28,29]. While this association was only significant in female subgroups, this may be explained by sex differences in spinal curvature and pelvic structure. Females typically have a greater lumbar lordosis and a wider pelvis [30], which may hinder proper spinal alignment on a firm mattress, whether supine or lateral, thereby increasing cervical load. Additionally, reverse causation cannot be ruled out: individuals with existing neck pain may intentionally select firmer mattresses based on the belief that they provide better spinal support.

Non-walking modes of commuting were associated with increased odds of NP. During driving or cycling, the body often maintains fixed or repetitive postures of forward leaning, head elevation, or torsion. Coupled with frequent whole-body vibration, this can lead to static contraction of neck and shoulder muscles and abnormal pressure distribution on intervertebral discs [31,32]. For passengers, passive body sway during travel, combined with the common habit of looking down at a smartphone while seated, may impose dynamic and destabilising loads on the cervical spine. Although the postural demands differ between drivers/passengers and cyclists, all three non-walking modes involve sustained or repetitive cervical spine loading that is largely absent during walking. We observed an association between commuting modes only in males, possibly because women often carry single-strap shoulder or crossbody bags while walking, both of which were independently associated with higher NP odds in our study and may offset the benefits of walking. This aligns with the biomechanical principle of symmetrical load carriage for maintaining spinal balance [33]. Unilateral loading cannot only cause tension imbalance and fatigue in muscles such as the trapezius and levator scapulae but may also, over the long term, induce compensatory postural scoliosis, leading to uneven stress on cervical facet joints and intervertebral discs [34,35].

Being female was associated with higher NP odds, consistent with previous studies [36–38]. This disparity may be attributed to a combination of factors including hormonal differences [39], muscle strength [40], pain sensitivity [41], occupational types, and societal roles. The association between age and NP reflects the impact of cervical spine degeneration on NP [36,42]. Higher mental workload intensity was associated with increased NP odds, consistent with previous findings [7]. This likely reflects that high-mental-workload individuals in our study were predominantly office workers, whose sedentary behaviour and computer use increase cervical spine burden. Studies have found an association between low educational attainment and higher odds of NP [36,43]. However, our study did not confirm such a correlation, which may be attributed to differences in the study population, geographical region, and other factors.

### Strengths and limitations

The multicentre design, including six Chinese cities, enhances population representativeness. The protocol underwent expert review and pilot testing, with unified investigator training and a multi-layer verification mechanism during data collection. These measures minimised information bias and ensured data quality. Importantly, unlike previous studies, this study quantified dose-response thresholds, which may inform clinical practice.

This study has several limitations. The cross-sectional design precludes causal inference, and findings require validation in longitudinal studies. Reverse causation cannot be ruled out for some associations (*e.g.* firm mattress use). Data relied on participant recall and self-assessment, which are susceptible to recall and social desirability bias. NP was self-reported without clinical confirmation or validated instruments, and the definition did not capture severity, functional limitation, or chronicity. We derived the AIC-based cutoffs for continuous variables from the same data set used for association analysis, which may lead to optimistically fitted thresholds due to overfitting. Therefore, these cutoffs should be regarded as exploratory and hypothesis-generating rather than definitive clinical thresholds, and external validation in independent samples is needed before clinical application. We did not apply correction for multiple comparisons for more than 15 exposures or for the separate PSM analyses, increasing type I error risk; marginal associations (*e.g.* smartphone screen height, supine sleeping) should be interpreted with caution. We combined driving, cycling, and being a passenger as non-walking transportation modes due to sample size constraints, although these sub-modes may differ in biomechanical mechanisms. We developed a questionnaire for this study, which has not been formally validated, although it underwent expert review and pilot testing.

## CONCLUSIONS

We identified and quantified threshold effects of key factors associated with NP among Chinese adults. Identified associated factors included computer use time for work ≥2 hours/day, smartphone use time for work ≥3 hours/day, smartphone screen height below horizontal eye level, and a firm mattress, which were associated with increased odds of NP. Sleeping time ≥7 hours/day, supine sleeping and walking as the primary mode of transportation were associated with decreased odds of NP. These quantified thresholds offer actionable benchmarks for posture management of NP in clinical practice and public health interventions.

## Additional material


Online Supplementary Document

## Data Availability

**Data availability:** Data generated or analysed during the study are available from the corresponding author by request.
