# Supplementary material for: Posture management for neck pain: identification and quantification of associated factors based on a multicentre cross-sectional study in China
Source: J Glob Health. 2026 Aug 21;16:04240. doi: 10.7189/jogh.16.04240 (PMC13494847; doi:10.7189/jogh.16.04240)
Supplement: Supplementary file 1 [file jogh-16-04240-s001.pdf]

Supplement to: Lu G, Mao H, Chen X, Sun X, Liu Y, Ma M, Li J, Zhou S, Hu J, Ma G, Wang Z, Xiu X, Zhuang M, Tang B, Zhu L, Yu J. Posture management for neck pain: identification and quantification of associated factors based on a multicentre cross-sectional study in China. J Glob Health. 2026;16:04016.

Questionnaire URL

The full electronic questionnaire used in this study is accessible at:

http://www.mskhealthcenter.com:9091/ches/index

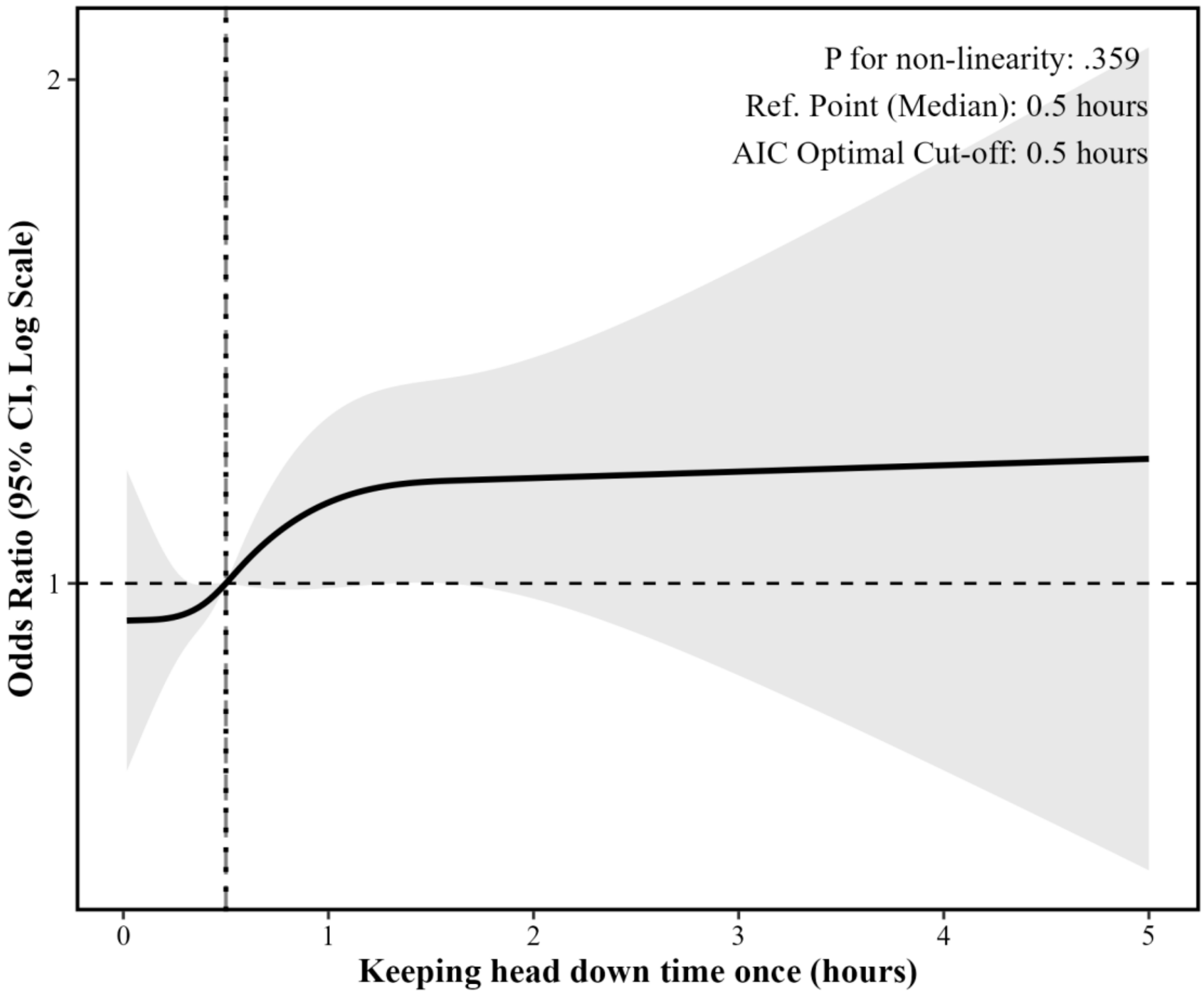

Figure S1. Restricted cubic splines analysis for keeping head down time once.

AIC, Akaike Information Criterion; CI, confidence interval.

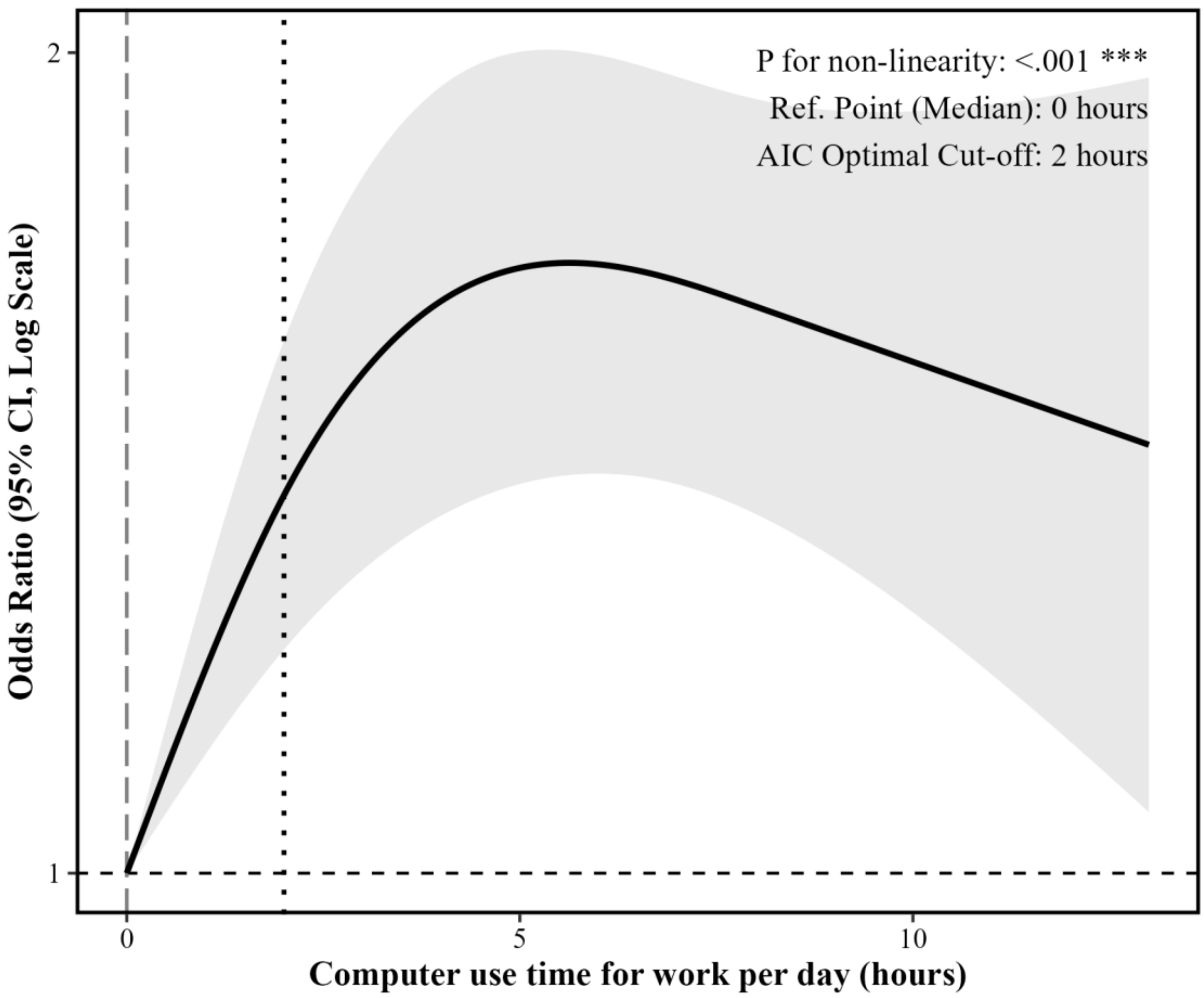

Figure S2. Restricted cubic splines analysis for daily computer use time for work.

AIC, Akaike Information Criterion; CI, confidence interval.

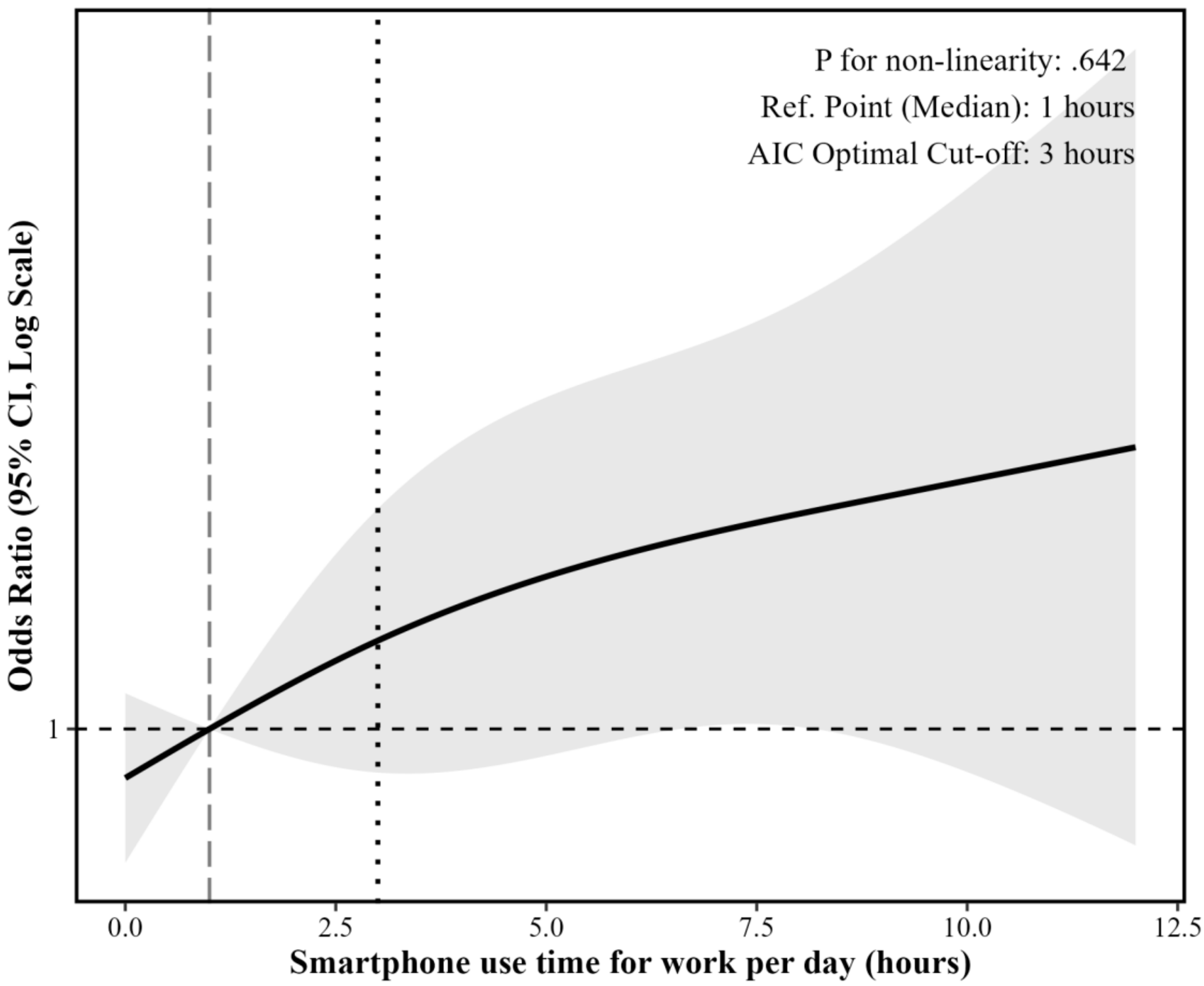

Figure S3. Restricted cubic splines analysis for daily smartphone use time for work. AIC, Akaike Information Criterion; CI, confidence interval.

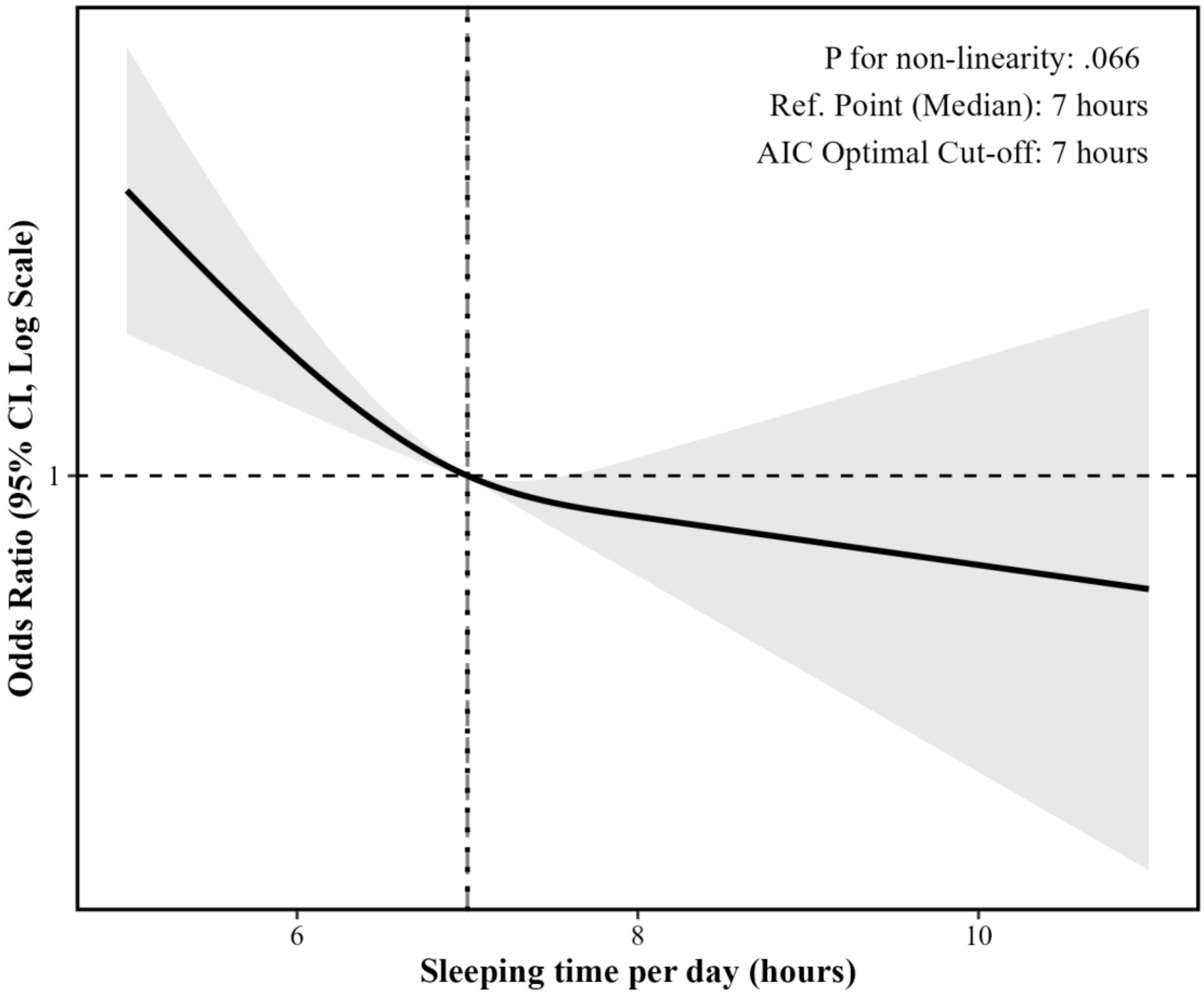

Figure S4. Restricted cubic splines analysis for daily sleep duration. AIC, Akaike Information Criterion; CI, confidence interval.

Table S1. Subgroup analysis

Characteristic	Subgroup	N	OR (95% CI)	*P* for interaction
Computer use time for work ≥2h/day				
	Sex			.855
	Male	2961	1.74 (1.38-2.21) ***	
	Female	4008	1.41 (1.17-1.70) ***	
	Age (years)			.270
	18-	4072	1.67 (1.41-1.98) ***	
	45-	2105	1.28 (0.93-1.77)	
	≥65	792	0.77 (0.28-1.96)	
Smartphone use time for work ≥3h/day				
	Sex			.225
	Male	2961	1.08 (0.88-1.33)	
	Female	4008	1.17 (0.99-1.38)	
	Age (years)			.235

	18-	4072	1.21 (1.02-1.42) *	
	45-	2105	1.06 (0.82-1.36)	
	≥65	792	1.31 (0.79-2.20)	
Smartphone screen height below horizontal eye level				
	Sex			.128
	Male	2961	1.26 (1.05-1.51) *	
	Female	4008	1.49 (1.29-1.73) ***	
	Age (years)			.005
	18-	4072	1.28 (1.10-1.48) **	
	45-	2105	1.44 (1.17-1.78) ***	
	≥65	792	1.53 (1.06-2.22) *	
Sleeping time ≥7h/day				
	Sex			.943
	Male	2961	0.77 (0.64-0.92) **	
	Female	4008	0.79 (0.68-0.91) **	
	Age (years)			.476

	18-	4072	0.82 (0.70-0.96) *	
	45-	2105	0.74 (0.61-0.89) **	
	≥65	792	0.65 (0.47-0.91) *	
Supine sleeping				
	Sex			.676
	Male	2961	0.82 (0.68-0.98) *	
	Female	4008	0.85 (0.73-0.98) *	
	Age (years)			.304
	18-	4072	0.90 (0.78-1.04)	
	45-	2105	0.77 (0.62-0.96) *	
	≥65	792	0.95 (0.63-1.44)	
Firm mattress				
	Sex			.018
	Male	2961	1.02 (0.85-1.22)	
	Female	4008	1.28 (1.11-1.49) ***	
	Age (years)			.157

	18-	4072	1.15 (0.99-1.33)	
	45-	2105	1.32 (1.07-1.62) *	
	≥65	792	0.86 (0.60-1.23)	
Walking as the primary mode of transportation				
	Sex			.002
	Male	2961	0.61 (0.50-0.74) ***	
	Female	4008	0.92 (0.80-1.06)	
	Age (years)			.176
	18-	4072	0.77 (0.66-0.90) ***	
	45-	2105	0.69 (0.56-0.85) ***	
	≥65	792	1.08 (0.75-1.55)	

Note: Data are OR (95% CI). * P<0.05, ** P<0.01, *** P<0.001.

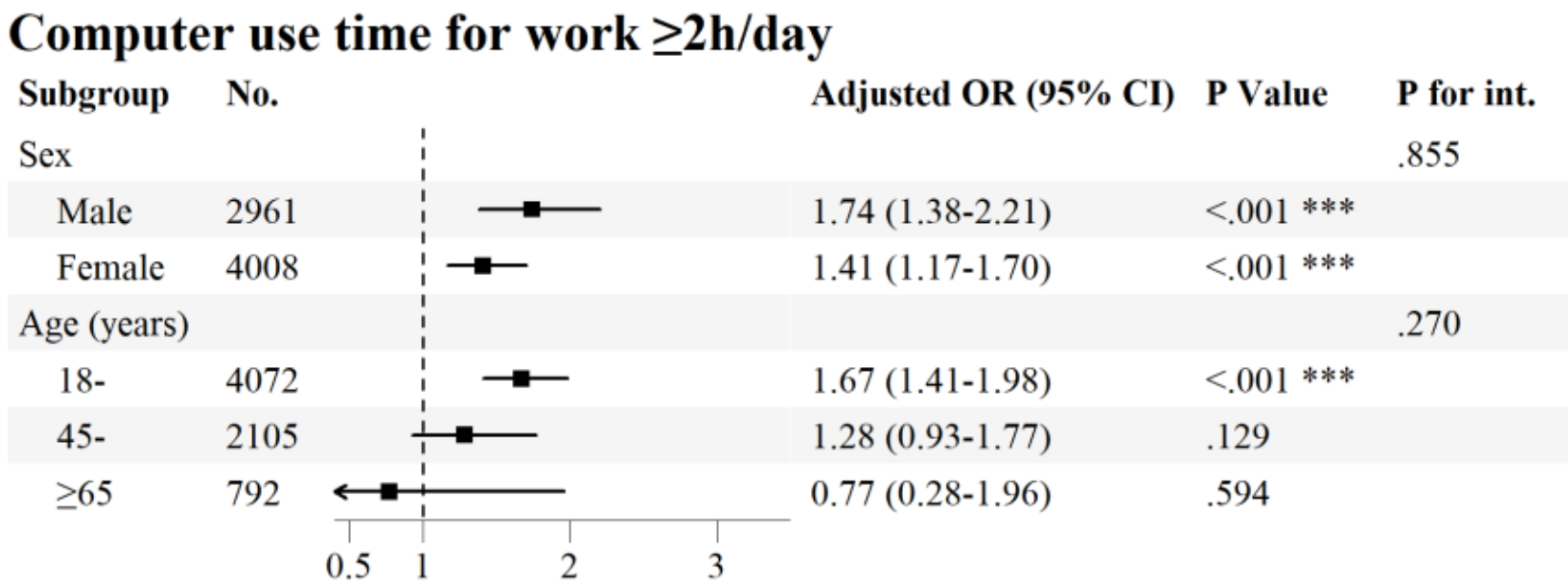

Figure S5. Association between computer use time for work ≥2h/day and neck pain across sex and age subgroups. CI, confidence interval; OR, odds ratio.

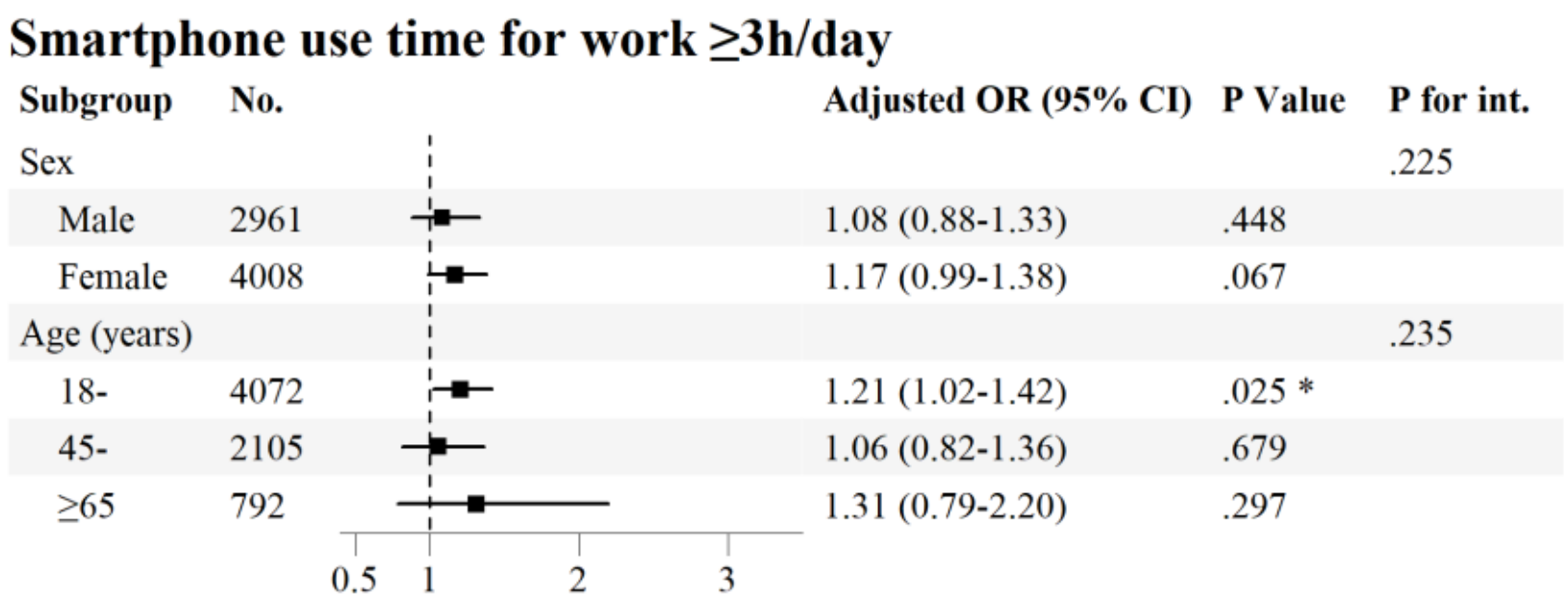

Figure S6. Association between smartphone use time for work ≥3h/day and neck pain across sex and age subgroups. CI, confidence interval; OR, odds ratio.

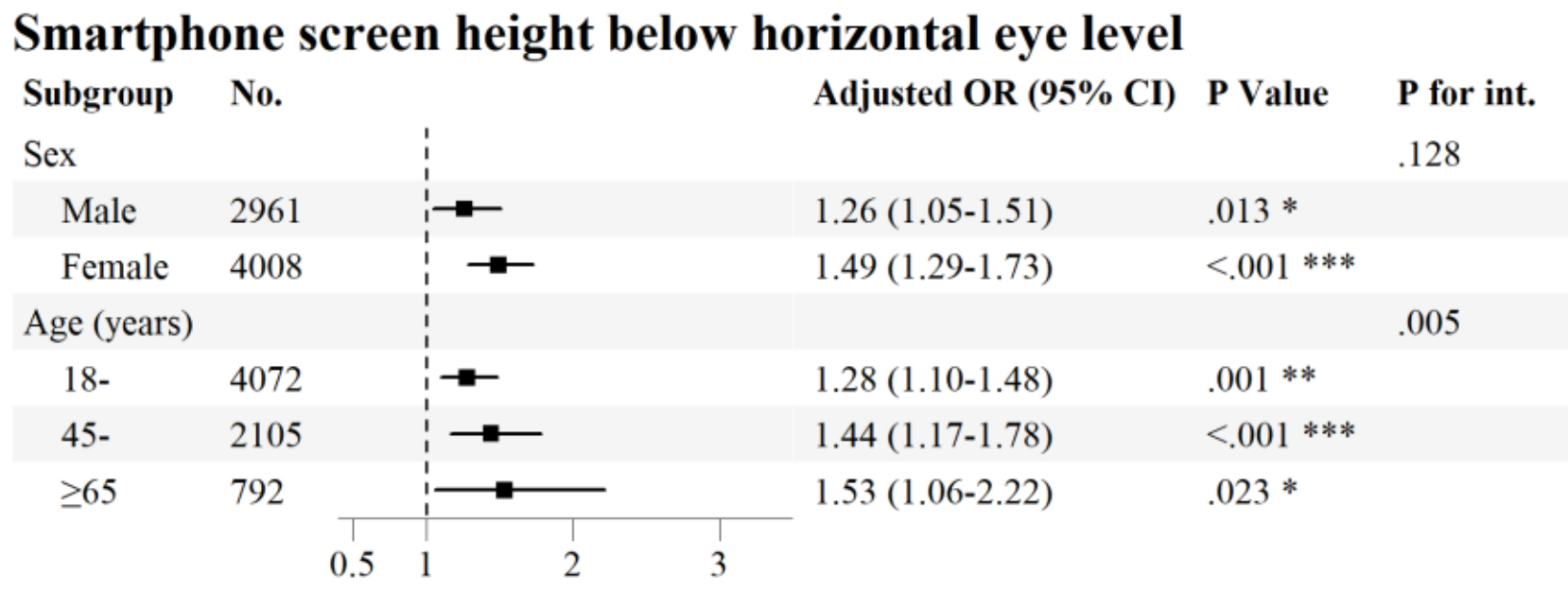

Figure S7. Association between smartphone screen height below horizontal eye level and neck pain across sex and age subgroups. CI, confidence interval; OR, odds ratio.

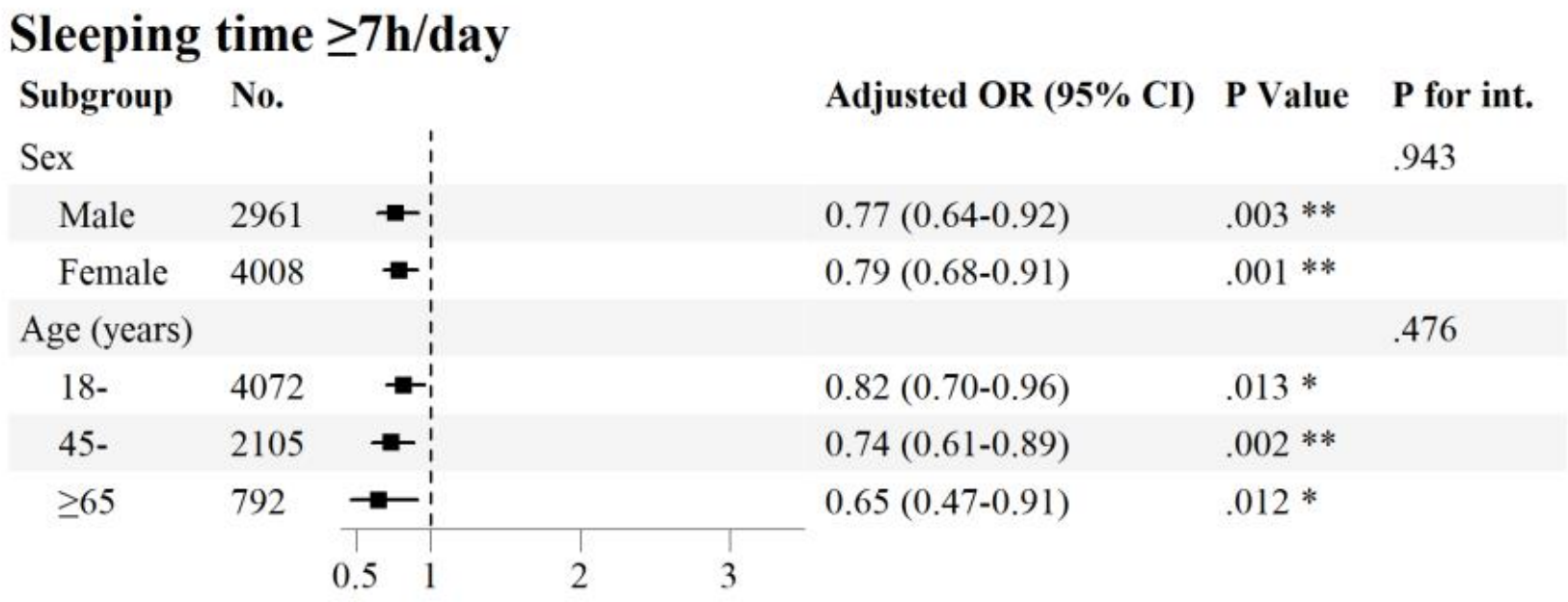

Figure S8. Association between sleeping time ≥7h/day and neck pain across sex and age subgroups. CI, confidence interval; OR, odds ratio.

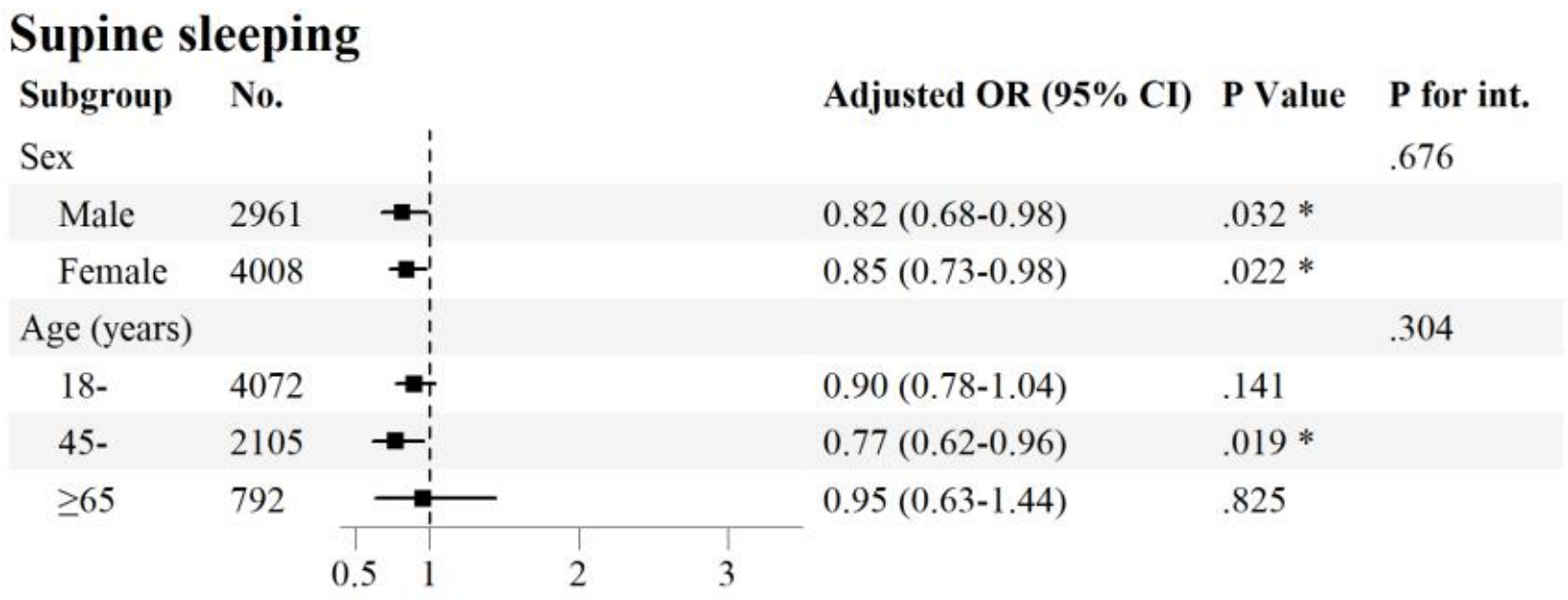

Figure S9. Association between supine sleeping and neck pain across sex and age subgroups. CI, confidence interval; OR, odds ratio.

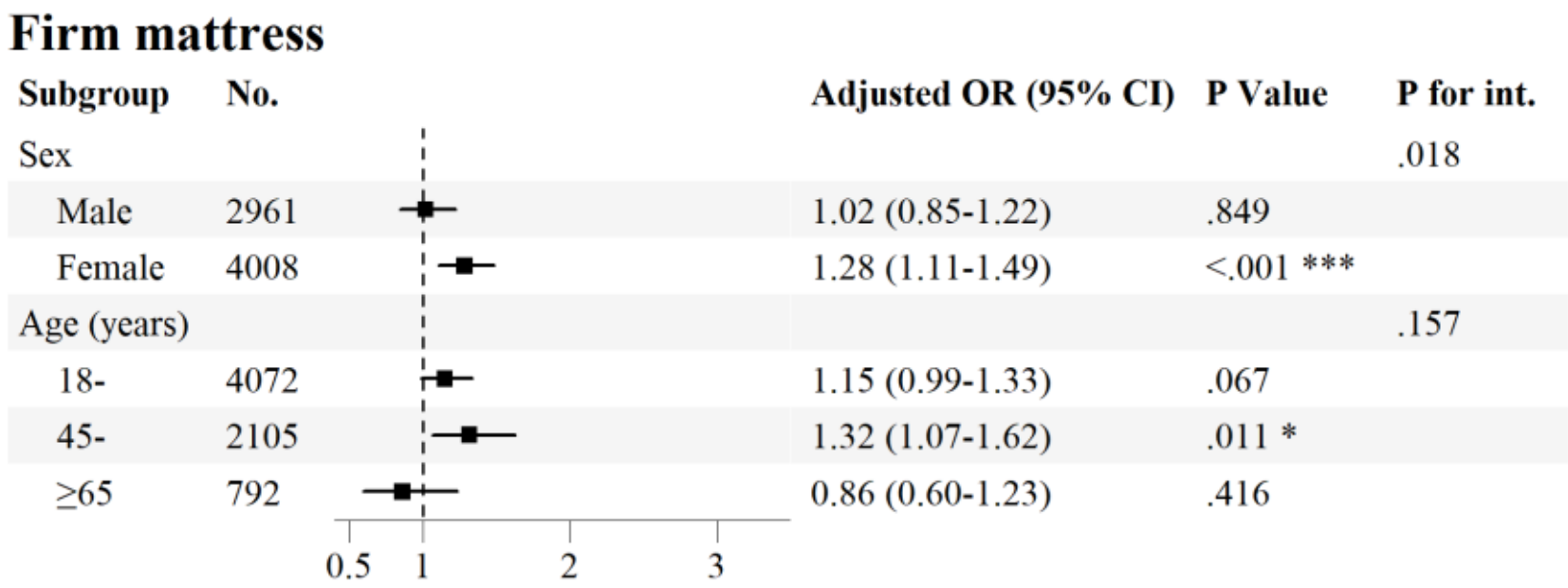

Figure S10. Association between firm mattress and neck pain across sex and age subgroups. CI, confidence interval; OR, odds ratio.

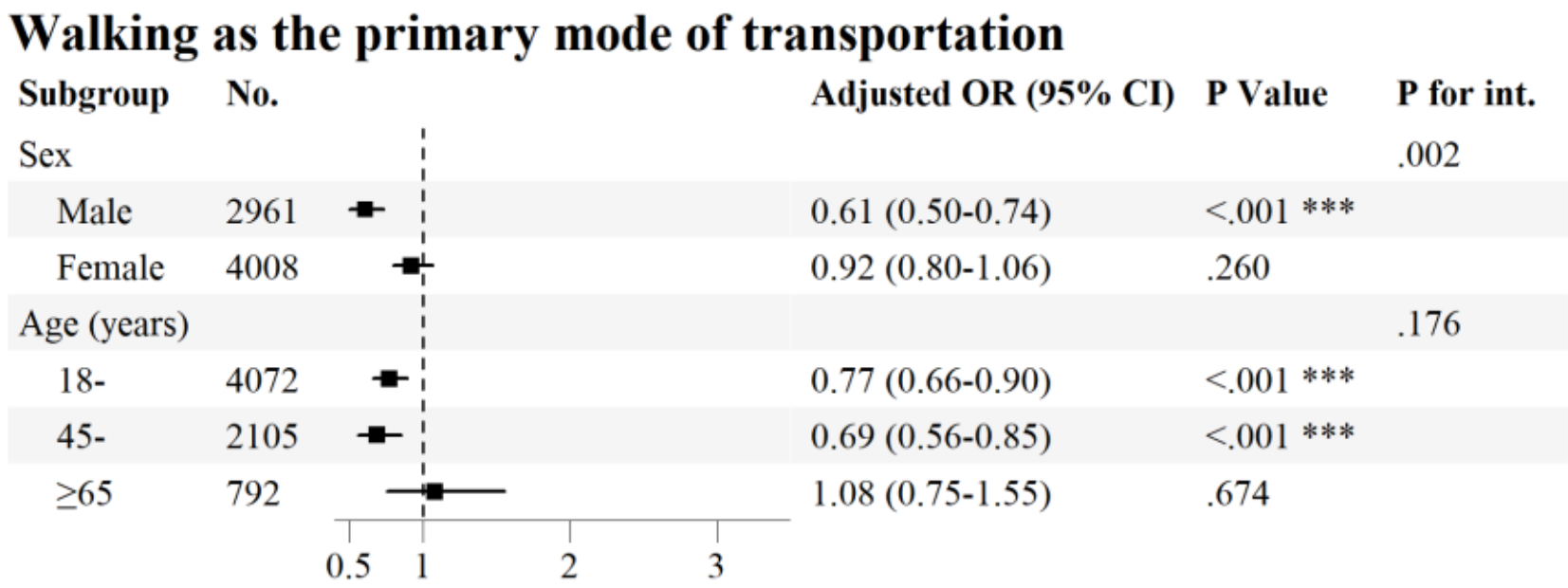

Figure S11. Association between walking as the primary mode of transportation and neck pain across sex and age subgroups. CI, confidence interval; OR, odds ratio.
